# Multitemporal Modelling of Socio-Economic Wildfire Drivers in Central Spain between the 1980s and the 2000s: Comparing Generalized Linear Models to Machine Learning Algorithms

**DOI:** 10.1371/journal.pone.0161344

**Published:** 2016-08-24

**Authors:** Lara Vilar, Israel Gómez, Javier Martínez-Vega, Pilar Echavarría, David Riaño, M. Pilar Martín

**Affiliations:** 1 Institute of Economics, Geography and Demography, Centre for Human and Social Sciences, Spanish National Research Council (CSIC), Madrid, Spain; 2 Institute of Geosciences, Spanish National Research Council-University Complutense of Madrid, Madrid, Spain; 3 Center for Spatial Technologies and Remote Sensing (CSTARS), University of California Davis, Davis, CA, United States of America; University of Maryland at College Park, UNITED STATES

## Abstract

The socio-economic factors are of key importance during all phases of wildfire management that include prevention, suppression and restoration. However, modeling these factors, at the proper spatial and temporal scale to understand fire regimes is still challenging. This study analyses socio-economic drivers of wildfire occurrence in central Spain. This site represents a good example of how human activities play a key role over wildfires in the European Mediterranean basin. Generalized Linear Models (GLM) and machine learning Maximum Entropy models (Maxent) predicted wildfire occurrence in the 1980s and also in the 2000s to identify changes between each period in the socio-economic drivers affecting wildfire occurrence. GLM base their estimation on wildfire presence-absence observations whereas Maxent on wildfire presence-only. According to indicators like sensitivity or commission error Maxent outperformed GLM in both periods. It achieved a sensitivity of 38.9% and a commission error of 43.9% for the 1980s, and 67.3% and 17.9% for the 2000s. Instead, GLM obtained 23.33, 64.97, 9.41 and 18.34%, respectively. However GLM performed steadier than Maxent in terms of the overall fit. Both models explained wildfires from predictors such as population density and Wildland Urban Interface (WUI), but differed in their relative contribution. As a result of the urban sprawl and an abandonment of rural areas, predictors like WUI and distance to roads increased their contribution to both models in the 2000s, whereas Forest-Grassland Interface (FGI) influence decreased. This study demonstrates that human component can be modelled with a spatio-temporal dimension to integrate it into wildfire risk assessment.

## Introduction

Wildfires have become an anthropic factor of regular and intense occurrence in the European Mediterranean basin [1]. In this region, humans cause over 90% of fire ignitions in connection to societal changes, Land-Cover and Land-Use Changes (LULC), and forest resources use [2]. European rural areas suffered a massive rural depopulation after Second World War, being more intense during the 1950s-1960s in southern European countries. In the 1980s, rural areas experienced new transformations due to agricultural modernization, development of construction industry, and increase in tourism [3]. Second/vacation homes also spread urban spaces over agricultural and natural areas. This trend persisted in the 2000s, with the proliferation in urbanization and infrastructure development [4, 5]. All these socio-economic changes influenced wildfire regime by altering their frequency, extent, intensity, severity and seasonality [6]. The most important ones are: (1) The rural depopulation that triggered the abandonment of arable lands, the growth of unmanaged shrubs and fuel accumulation. The combination of unfavorable weather conditions together with these higher fuel loads translated into larger wildfires [7]; (2) The expansion of the Wildland Urban Interface (WUI) due to urban development close to natural areas. Fire ignition and propagation risk increased due to this higher human pressure on natural areas [8]; (3) The larger number of visitors to the natural areas for tourism and recreational activities [2]. Either by negligence or arson, these practices caused more human-induced wildfires [9]; (4) The use of fire as a traditional tool for agriculture and cattle grazing [2]. Its application to eliminate harvest waste and to clear brushwood in the croplands boundaries or in abandoned agricultural land caused fire spread into neighbor natural areas. Likewise, controlled fires to regenerate herbaceous vegetation and eliminate shrubs for cattle grazing sometimes went wild.

The human impact on the wildfires regime is difficult to model since it requires the identification, quantification and mapping of behavioral factors [10]. Nevertheless, several studies incorporate these human and socio-economic drivers among other physical variables to predict wildfire occurrence by using diverse statistical methods. For example, Generalized Linear Models (GLM) showed suitable results in areas with Mediterranean conditions like California [11, 12] or Spain [13, 14]. Among GLM, presence-absence models such as the logistic regression, can handle the unbalanced sample of rare wildfire presences versus the common wildfire absences. Machine learning algorithms e.g. random forest [15, 16], classification trees [17] and weights of evidence [18] can also properly predict and explain wildfire occurrence. One of the advantages of these algorithms is that they are non-parametric models. Therefore, the input explanatory variables interrelations are not defined a priori, but rather derived from iterative training and testing using random data subsets [19].

Among the existing machine learning tools, the presence-only Maximum Entropy (Maxent) algorithm frequently builds animal and plant species Spatial Distribution Models (SDM) in relation to environmental variables [16, 20]. Especially for small sample sizes, Maxent demonstrates higher prediction accuracy than other SDM [21, 22]. Because fire ignition distribution can be compared to species distribution, some authors apply Maxent to model environmental drivers related to the spatial variability in wildfire probability [23, 24]. Similarly to wildlife studies [21, 25], Bar Massada et al. [16] found that the algorithm chosen affects the wildfire occurrence model. They tested one statistical and two machine-learning models to render a similar performance and selection of variables, but the output Maxent map of predicted wildfire probabilities was markedly different.

Independently of the variables and models applied, most wildfire modeling studies select specific time periods to which the explicative variables are referred to. Multitemporal analysis mainly focuses on either climatic and/or LULC and their relationship with wildfire occurrence at different spatial scales [26–30]. However, changes in wildfire regime are also related to changes in other socio-economic drivers like population, unemployment, etc. In this context, this study (1) analyzes changes in multiple socio-economic drivers of wildfire occurrence by building and comparing models for the 1980s and the 2000s; and (2) evaluates the predictive as well as explicative performance of a presence-absence GLM and a presence-only Maxent wildfire occurrence probability model in order to analyze the impact on the results of the model selection.

## Materials and Methods

### Study site

**Fig 1:**
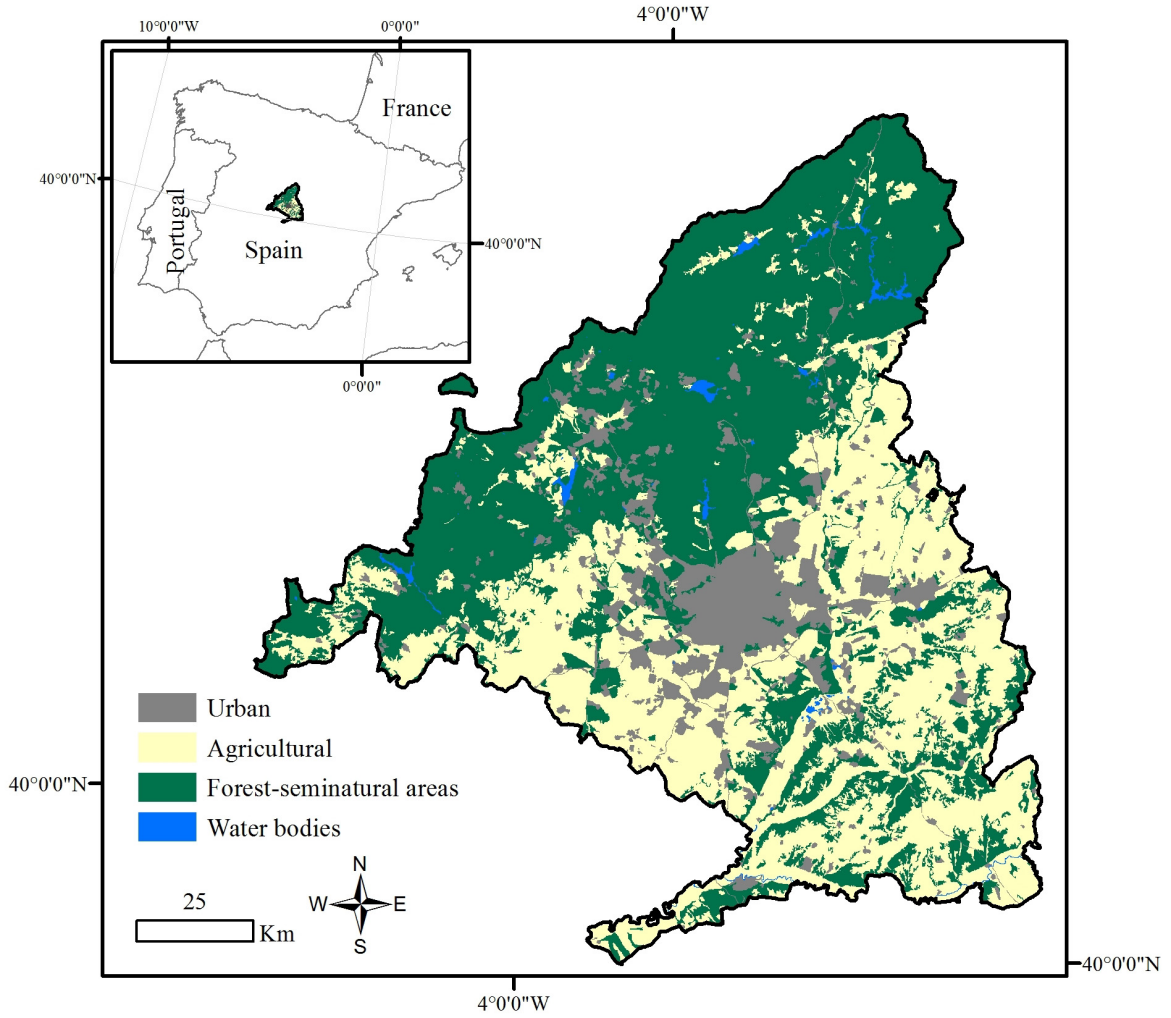
CORINE Land Cover (CLC, http://www.eea.europa.eu/publications/COR0-landcover) reclassified map for the Madrid region study site in 2000.

Similarly to other European Mediterranean areas, Madrid region (Fig 1) experienced an important socio-economic transformation in the last decades. Its ~8028 km^2^ occupies ~1.6% of the Spanish territory and contains a northeast-southwest mountain range [31]. This orographic barrier, together with its further distance to the sea, causes colder winters and warmer summers, and hence higher thermal amplitude (20°C) than coastal Mediterranean regions (10–15°C) [32]. Annual precipitation oscillates between 350 mm in the basin and 600 mm in the sierras [33]. Oak forest (*Quercus pyrenaica*) are typical in the sierras at 1200–1700 m elevations. In more humid areas, oaks combine with other broadleaved species like rowan (*Sorbus aucuparia*) or narrow-leafed ash (*Fraxinus angustifolia*). Among others, common shrubs here are broom (*Cytisus scoparious*), bridal broom (*Retama monosperma*) and hawthorn (*Crataegus monogyna*). At lower elevations, laudanum (*Cistus ladanifer*) becomes more abundant. In areas where precipitation is higher, beech (*Fagus sylvatica*) substitutes the oak forest, together with chestnut (*Castanea sativa*) and silver birch (*Betula pendula*) (Consejería de Medio Ambiente y Desarrollo Regional, CMADR, http://www.madrid.org/).

Madrid region is one of the most populated areas in Spain with ~6.4 million inhabitants in 2015 (http://www.ine.es/) and a population density of ~800 inhabitants/km^2^ (http://www.madrid.org/). The decadal population growth rate was 4.67, 9.62 and 18.41% in 1981–1991, 1991–2001 and 2001–2011, respectively (http://www.ine.es/). During the 1980s, depopulation began in rural areas whereas urban areas grew 50%, mainly thanks to the rise in small and medium cities close to Madrid. Urban areas spread over agricultural and natural areas due to the social demand for first and second/vacation homes [34]. This process continued into the 2000s. Socio-economic changes mainly implied (1) the abandonment of rural areas and hence of the traditional activities related to agriculture and livestock; (2) the increase in human pressure over natural areas due to tourism, recreational use, and urban expansion; and (3) the development of transport networks. Human activities cause ~90% of wildfires, as they occur close to roads, railways, landfills and the WUI [35]. In fact, the WUI is one of the major concerns for fire managers in this region [10], as is the case in other Mediterranean areas worldwide [12, 36].

### Wildfire occurrence data

Landsat satellite series offer a homogeneous and coherent temporal coverage at 30 m spatial resolution since 1982. After searching for cloud free images, the 1980s period included in the analysis the years 1985, 1987, 1988, 1989, 1991 from Landsat 5 Thematic Mapper (TM) images whereas the 2000s contained the years 2000–2005 from Landsat 7 Enhanced Thematic Mapper Plus (ETM+). The combination of a Burnt Area Index (BAIM) [37] value >100 and visual interpretation of 7-4-1 and 7-5-4 RGB color band composites delimited the wildfire perimeters. These composites successfully discriminated burned areas from these satellites on previous studies [38–40]. In addition, the Spanish National Wildfire records database from the Spanish Ministry of Agriculture, Food and Environment, registers the fire location both referred to municipalities (NUTS5) and 10 km^2^ grid cells. To confirm the fire perimeters were not false alarms, further analysis considered only those Landsat perimeters that spatially matched the wildfires occurred according to the official database in the NUTS5 or 10 km grid cells. In order to obtain a common spatial reference unit, those confirmed Landsat perimeters were referred to the 1 km^2^ European Grid System (INSPIRE, http://www.eea.europa.eu/data-and-maps/data/eea-reference-grids-1). This conversion assigned to each grid cell a value of one for those cells partially or totally overlapping with a confirmed fire perimeter (N = 1) or a zero otherwise (N = 0).

### Socio-economic drivers

**Fig 2:**
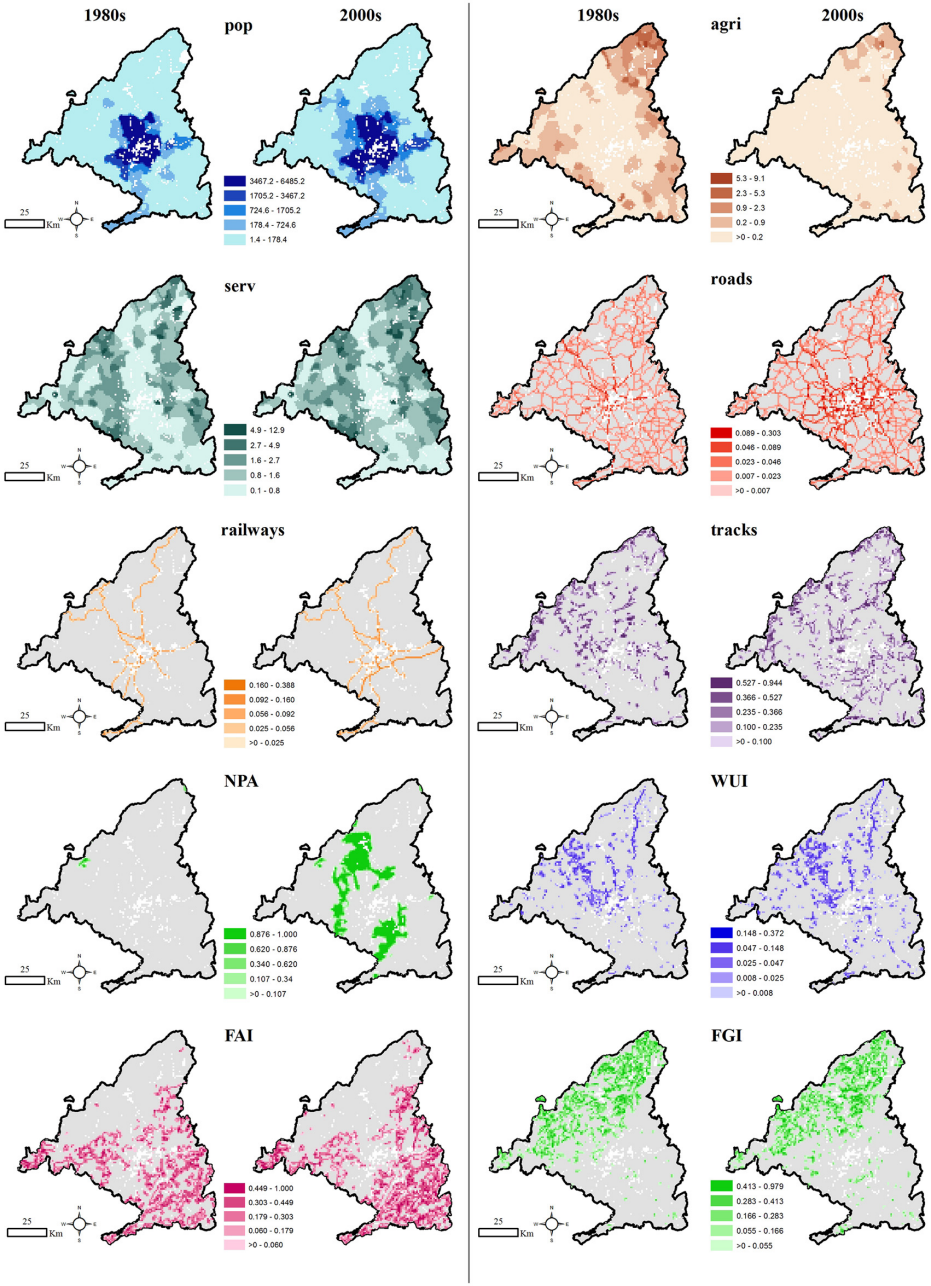
Socio-economic independent variables maps for the 1980s and 2000s excluding the non-natural vegetation CLC classes (white). Variables do not take place in some areas (grey).

Leone et al. [41], Vilar del Hoyo et al. [42], Martínez et al. [13] and Vilar del Hoyo et al. [43] describe the socio-economic independent variables of human-caused wildfires in European Mediterranean environments. Among them, the ones selected here include those that can be spatially represented and more directly linked to wildfire occurrence (Table 1). Depending on the source and typology of each variable, either ArcMap 10 [44], or Python 2.6 with ESRI ArcPy libraries processed them to match the INSPIRE grid for the two time periods (Fig 2). Population density (*pop*), density of agriculture workforce (*agri*) and density of services workforce (*serv*) independent variables came from a statistical source. Since their spatial unit was NUTS5, their statistical value was assigned to the corresponding INSPIRE cell. When a cell included several NUTS5 polygons, the cell contained a weighted average by the area occupied by each polygon [43]. Cartographic sources provided independent variables such as *roads*, *railways*, *tracks*, *WUI*, *Forest-Agricultural Interface* (*FAI*), *Forest-Grassland Interface* (*FGI*) and *Natural Protected Areas* (*NPA*). Spatial crossing converted the *NPA* polygons to the INSPIRE grid. The rest of these variables were transformed from lines to buffers of influence. These buffers, indicated in the second column of Table 1, define the minimum distance from those lines that the national and regional legislation requires to keep clean to prevent wildfires, see statutory order 31/2003 [45]. After assigning these buffer polygons to the INSPIRE grid, this approach obtained density values for all the socio-economic independent variables (Fig 2).

**Table 1:** Selected socio-economic independent variables of wildfire occurrence.

Independent variables acronym	Description (years)		Relation to wildfire occurrence	Data source
*pop*		Population density (1981, 2001)	A population density increase leads to higher human pressure over natural areas that can cause wildfires due to accidents or negligence	NUTS5 Population census^1^^,^^2^
*agri*		Agriculture workforce density (1981, 2001)	An agricultural workers decrease relates to the abandonment of traditional activities in rural areas that increases fuel loads available to burn.	NUTS5 Population census^1^
*serv*		Services workforce density (1981, 2001)	A services workforce density increase indirectly relates to more recreational activities that can cause wildfires due to accidents or negligence	NUTS5 Population census^1^
*roads*		15, 25 and 50 m road buffers (1982, 2000)	Fire ignitions are more likely close to roads due to accidents or negligence like cigarette butts, while human pressure increases as a result of easier access to natural areas	1:50,000 Topographic map^3^
*railways*		70 and 100 m railway buffers (1982, 2000)	Fire ignitions are more likely close to railways due to accidents, like train braking sparks or negligence, while human pressure increases as a result of easier access to natural areas	1:50,000 Topographic map^3^
*tracks*		300 m tracks buffer (1982, 2000)	Fire ignitions are more likely close to tracks due to accidents or negligence, like cigarette butts, while human pressure increases as a result of easier access to natural areas	1:50,000 Topographic map^3^
*NPA*		NPA (1982, 2000)	Natural areas protection can reduce wildfires but can also promote them due to social unrest against the restriction of some activities	1:50,000 NPA map^3^
*WUI*		12.5m WUI buffer (1982, 2000)	WUI increases human pressure on natural areas that can cause wildfires due to accidents and negligence	1:50,000 Vegetation and land use map^3^; 1:100,000 CLC map^4^
*FAI*		200 m FAI buffer (1982, 2000)	Agricultural activities in the FAI that use fire to eliminate harvest waste and to clear brushwood in the croplands boundaries can spread fire into neighbor natural areas	1:50,000 Vegetation and land use map^3^; 1:100,000 CLC map^4^
*FGI*		200 m FGI buffer (1982, 2000)	Controlled fires in the FGI to regenerate herbaceous vegetation and eliminate shrubs for cattle grazing can go wild	1:50,000 Vegetation and land use map^3^; 1:100,000 CLC map^4^

^1^. Instituto Nacional de Estadística: (INE, www.ine.es)

^2^. Instituto de Estadística Comunidad de Madrid (IE, www.madrid.org/iestadis/)

^3^. Consejería de Medio Ambiente y Desarrollo Regional (CMADR, www.madrid.org)

^4^. CORINE Land Cover (CLC, www.eea.europa.eu/publications/COR0-landcover)

The relation between wildfire occurrence and the socio-economic variables excludes from their models non-natural vegetation CLC classes, such as artificial, bare rocks, water bodies, inland marshes and agriculture. This analysis assumes that wildfires are not possible in marshes due to the water submerged vegetation or in artificial, water and bare rocks classes because of the absence of vegetation. Fires can start in the CLC agriculture class, but they are considered wildfires as long as they do affect natural vegetation. Even though mainly agricultural land occupies the pasture CLC class, it was still included in the analysis as it contains significant areas of natural vegetation and agro-forestry.

### Wildfire occurrence probability models

GLM and Maxent modelled wildfire occurrence probability in the 1980s and 2000s separately in relation to the explanatory socio-economic variables in Table 1 and Fig 2. GLM are extensions of linear regression models that support dependent variables with non-normal distributions, such as binomials [46, 47]. The model assumes that the independent variables are not correlated, since multicollinearity inflates the variance amongst them [48]. For this reason, Spearman correlations checked the multicollinearity among independent variables and Variance Inflation Factor (VIF) detected the degree of multicollinearity when predictors are not centered [49]. If Spearman correlations were >0.6 and/or VIF>10 [50], multicollinearity existed and one of the independent variables was excluded from the analysis.

To balance the presences/absences, only a small subset of the INSPIRE cells that contained wildfire absences was selected to match the number of presences. In previous works, cells with no wildfire were randomly chosen [51–54]. This introduces in the model a deterministic offset term of—log (π) that does not bias the analysis [10]; where π denotes the response-specific sampling rate. When N = 1, π is also 1, and when N = 0, π = π. After analyzing the error term minimization, 20% of the wildfire absence cells were considered an appropriate subset. The resulting dataset was divided in two groups, 75% for model calibration and 25% for validation. The lowest Akaike’s Information Criterion (AIC) value selected the best model. Adding independent variables step by step assessed their individual contribution to the model performance. The estimated model coefficients were analyzed and interpreted for each period.

Receiver Operating Characteristic (ROC) curve evaluated the models prediction accuracy [55]. The Area Under the Curve (AUC) represents the probability that a randomly chosen wildfire presence case exceeds the one of randomly choosing an absence. AUC ranks between 0 and 1; <0.5 means no discrimination; 0.5–0.69 poor; 0.7–0.79 reasonable; 0.8–0.89 excellent; and >0.9 exceptional. Additionally, a cross tabulation exercise checked the percentage of true (sensitivity) and false (commission error) wildfires presences, and true (specificity) and false (omission error) absences that the model classified correctly (true) or incorrectly (false).

In order to assign a probability of occurrence, Maxent finds the maximum entropy in the closest uniform distribution to the empirical data and assures a match between this distribution and the empirical average [20]. As a presence-only algorithm, wildfire absences are treated instead as background, where a wildfire would actually be possible if independent variables values were favorable [56]. Similarly to GLM, Maxent models were built using 75% of the wildfire occurrence cells. The remaining 25% and a set of 1600 random background cells independently validated the model. Excluding one variable at a time, Maxent jack-knife test evaluated the relative contribution in percentage of each variable to the model fitting [57–59]. Through the generation of response curves, Maxent illustrates how important each variable is at explaining wildfire occurrence and how much unique information each one provides [58]. As for GLM, ROC and AUC values assessed the Maxent models performance. Given the presence-only nature of Maxent, the cross tabulation exercise only calculated the sensitivity and commission error statistics as they are related to presence.

Models were fit using R 3.1.0 [60] with *car* [61], *mgcv* [62] and *ROCR* [63] packages and Maxent 3.3.3k freeware [20], http://www.cs.princeton.edu/~schapire/maxent/).

## Results

### GLM and Maxent modelling

The exploratory analysis of the input data demonstrated only a high Spearman correlation (-0.69) for *agri* and *serv* independent variables in the 2000s. Since *serv* had a lower Pearson correlation to wildfire occurrence than *agri*, *serv* was eliminated from GLM for the 2000s period. In addition, the <10 VIF values ranging from 1.007 to 4.337 for all independent variables and both periods, indicated a lack of multicollinearity among them.

Table 2 indicates that the selected predictors in the fitted GLM model were all significant but different for each period. The model excluded *pop*, *roads*, *tracks and FAI* in the 1980s; *serv* in the 2000s; and *NPA and FGI* for either period. Wildfire occurrence in the 1980s was positively related to *WUI* and *railways* and, to a lesser extent, to *serv* whereas it was negatively related with *agri*. Regarding the 2000s, a strong positive relation was obtained for *WUI*, *roads* and *railways* whereas *agri* was again negatively related. For the 1980s, the model validation provided an AUC of 0.81 and a sensitivity, commission error, specificity and omission error of 23.33, 64.97, 35.03 and 76.67%, respectively. For the 2000s, AUC was 0.75, and sensitivity, commission error, specificity and omission error were 9.41, 18.34, 86.66 and 38.33%, respectively.

**Table 2:** Estimated coefficients and significances (Wald test) for each of the GLM predictors in the 1980s and 2000s. Coefficients indicate odds of a wildfire to happen.

	1980s		2000s	
Predictor	Estimated coefficient	Probability (>|z|)	Estimated coefficient	Probability (>|z|)
Intercept	-2.22295	< 2e-16 ***	-2.411	< 2e-16 ***
*pop*	-	-	3.645e-04	1.08e-08 ***
*agri*	-0.249	0.004**	-2.195e-03	7.07e-05 ***
*serv*	0.447	7.62e-09***	-	-
*roads*	6.966	0.07	10.76	1.72e-05 ***
*railways*	6.990	0.015*	5.109	0.025 *
*tracks*	-0.576	0.138	0.759	0.034 *
*NPA*	-	-	-	-
*WUI*	12.709	0.005**	11.23	0.0023 **
*FAI*	-	-	0.752	0.0291 *
*FGI*	-	-	-	-

Signif. codes: 0 ‘***’ 0.001 ‘**’ 0.01 ‘*’ 0.05 ‘.’ 0.1 ‘ ‘ 1

Table 3 shows that the predictor *pop* contributed the most to the Maxent model in both periods. In the 1980s, *FAI*, *FGI*, *agri* and *roads* followed *pop*, while *serv*, *roads*, *NPA* and *WUI* did in the 2000s. *Serv*, *railways*, *NPA* and *WUI* were more relevant in the 2000s than in the 1980s. The AUC was 0.70 for the 1980s and 0.74 for the 2000s, and sensitivity and commission error were 23.33 and 64.97% for the 1980s and 67.3 and 17.9% for the 2000s, respectively.

**Table 3:** Percent contribution of each predictor to the Maxent models in the 1980s and 2000s. In bold, the five variables with the highest contribution.

	1980s	2000s
Predictor	Contribution (%)	Contribution (%)
*pop*	**50.7**	**39.6**
*agri*	**5.6**	1.1
*serv*	2.5	**15.2**
*roads*	**13.8**	**8.8**
*railways*	2	4
*tracks*	4.1	4.3
*NPA*	0	**8.8**
*WUI*	2.1	**8.6**
*FAI*	**13.6**	6.4
*FGI*	**5.7**	3.3

**Fig 3:**
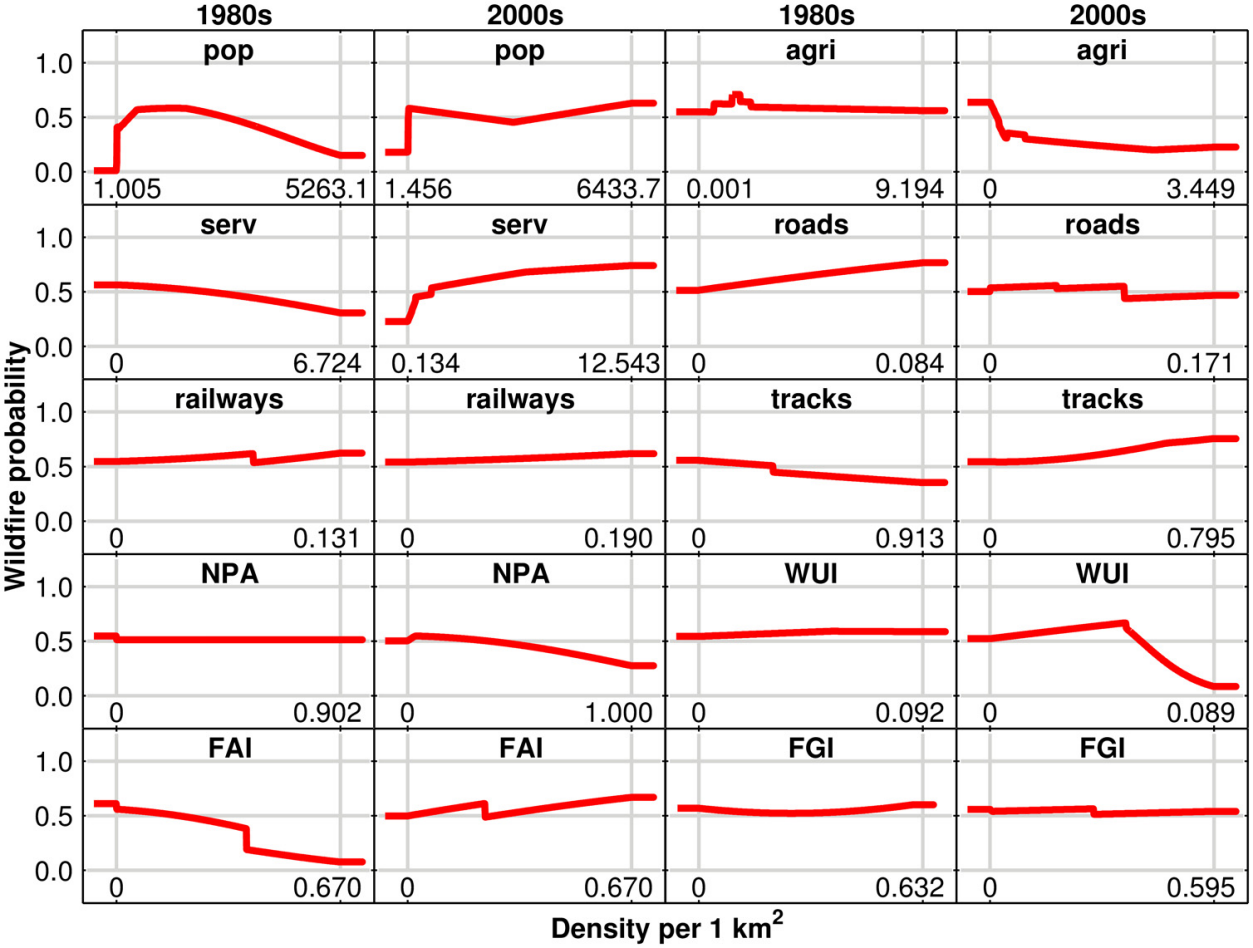
Curves indicate the mean wildfire predicted probability from Maxent in the 1980s and 2000s.

Maxent predicted wildfire probability varied from the 1980s to the 2000s (Fig 3). The probability reached a maximum for intermediate *pop* densities in the 1980s, but *pop* had a slightly positive influence after mid densities in the 2000s. The small positive influence in wildfire probability of *agri* as density increases in the 1980s opposes its negative influence in the 2000s. Regarding *serv*, Maxent predicted a negative influence in the probability across intermediate values in the 1980s, while it was positive in the 2000s. *Roads* had a positive influence on higher densities in the 1980s, while the positive influence was higher for lower densities in the 2000s. *Railways* tended to have a positive influence in both periods. For *tracks* and *FAI*, the variables had a negative influence with density in the 1980s, but it was the opposite in the 2000s. *NPA* tended to be stable in the 1980s, while it had a negative influence in the 2000s. In the case of *WUI*, it had a positive influence with density in the 1980s, whereas it first had a positive influence up to mid densities in the 2000s but it was negative afterwards. Wildfire probability slightly decreased with *FGI* density in the 1980s, while it remained stable across this variable in the 2000s.

**Fig 4:**
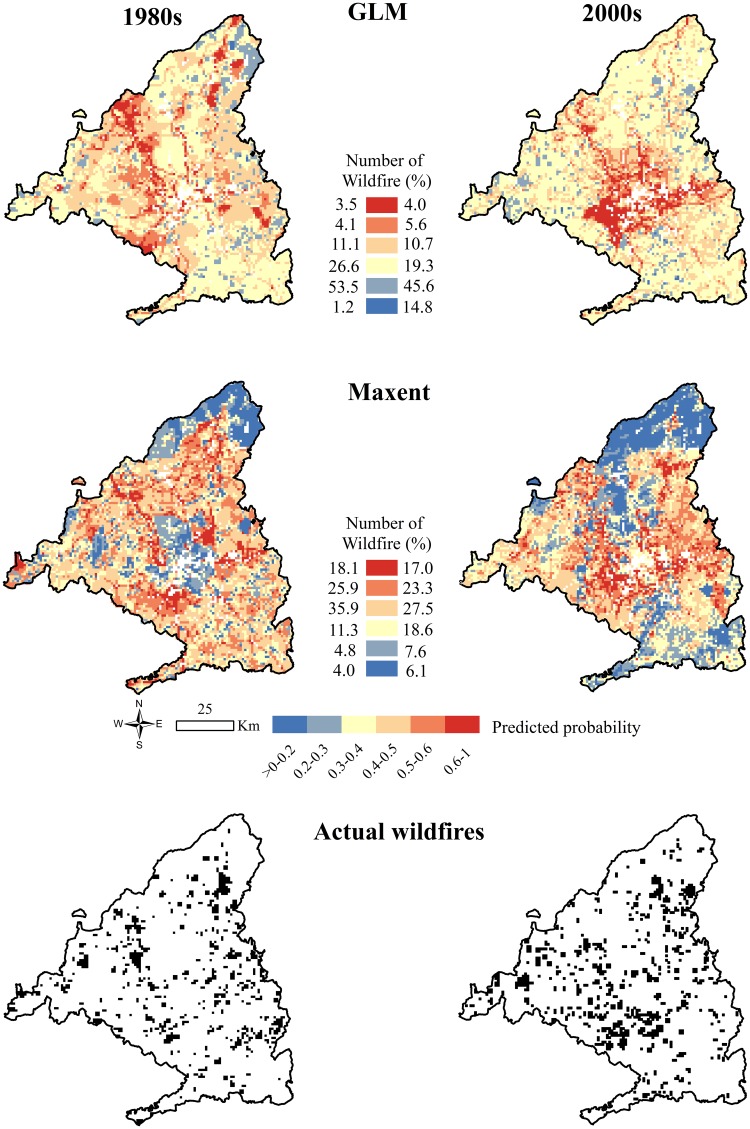
Wildfire predicted probability maps after applying GLM and Maxent models in the 1980s and 2000s and wildfire occurrence (black) in each period. White cells in GLM and Maxent maps represent the excluded cells from the analysis. Probability maps include a legend with the percentage of actual wildfires that occurred at each probability interval in the 1980s (left) and 2000s (right).

Fig 4 revealed different spatial patterns between GLM and Maxent models with a low Pearson correlation between them for both the 1980s (r = 0.38) and the 2000s (r = 0.40). In 1980s, cells in the GLM map with >0.5 probabilities were mainly located in the north and northwest, near *railways* and areas with higher *WUI*. Only 3.5% of actual wildfires matched high (>0.6) probability cells. In contrast, 53.5% of actual wildfires coincided with low (0.2–0.3) probability ones. The 2000s GLM map exhibits medium to high probabilities south of the main urban area (Madrid) and in a northeast corridor, near *roads*, *WUI* and cells with higher *pop*. The 2000s GLM performs slightly better since, even though still only 4.0% of actual wildfires matched high probability cells.

Maxent obtained similar probabilities for both periods (Fig 4). In the 1980s, cells with higher probability matched with higher *serv* and *pop* densities north, northwest and south of Madrid city. In this period, 18.1% of actual wildfires were in high probability cells and only 4% of them occurred in low probability ones. In the 2000s, cells with higher probability coincided, not only with higher *serv* and *pop* densities surrounding Madrid city, but also near to *roads*. In this case, 17.0% of actual wildfires were in high probability cells and 6.1% in low probability ones.

## Discussion

Multitemporal modelling of socio-economic wildfire drivers revealed that both GLM and Maxent models increased wildfire probability from the 1980s to the 2000s in the south of the main urban area. Building a model for each period provides additional information on the relative contribution of the predictors as indicators of changes in the region. In the last two decades, these areas have suffered an urban sprawl and consequent increase in WUI [64, 65]. The identification of key socio-economic drivers can help implement management plans to reduce their impact. Predictors like *WUI* and *railways* contributed positively to wildfire occurrence for both models and their relevance increased in the 2000s. The significance of *roads* in 2000s is coincident with Padilla and Vega-García [66] results that built a model for Spain from 2002 to 2005. Working also in Spain during an earlier period from 1988 to 2000, Martínez et al. [13] found that *roads* occupied only intermediate importance among the variables in their models. In a study at the Huron—Manistee National Forest, Michigan, USA, between 1994 and 2009, Bar Massada et al. [16] identified as the most important predictors, distance to nearest road, distance to structure and structure density, where a structure was either a house or non-residential building. Other transport networks such as *tracks* and *railways* increased their importance in the 2000s for both models, which it is also in tune with the socio-economic development of the region and its positive relation to wildfire occurrence. Regarding *WUI*, it had the highest contribution to the GLM in both periods. It was also part of the Maxent model but not in the top three. This predictor had a notable contribution to Rodrigues et al. [67] model for the Madrid region in 1988–2011. In Chuvieco et al. [14], it also largely contributed to wildfire occurrence in this region for 1990–2004. After testing both natural and socio-economic drivers, Syphard et al. [11] found that *WUI* along with *pop* explained the greatest amount of variability in California from 1960 to 2000.

The high Spearman correlation between *agri* and *serv* predictors in the 2000s excluded *serv* from the GLM. Since it was negative, cells with high *agri* had low *serv* and vice-versa. This indicates a segregation of these workforces between cells in this period but more mingled in the 1980s. Removing *serv* from the 1980s GLM increased AUC in ~1%, whereas it kept the same AUC in the 1980s and decreased ~3% in the 2000s for Maxent. However, it was the second largest contributor for Maxent in the 2000s. This result suggests that the increase in *serv* could cause more wildfires due to more recreational activities that pressure natural areas or to the above mentioned urban growth. Nevertheless, the relevance of this predictor requires further investigation on other study sites to confirm or to discharge this hypothesis, as a coincidental indirect indicator of wildfire occurrence.

The importance of *FGI* decreased dramatically in the 2000s for both models. This fact indicates less use of fire to gain or maintain herbaceous vegetation for cattle grazing. In addition, *agri* had a negative impact for GLM and decreased its contribution from 1980s to 2000s for both models. This result is contrary to the a-priori expectation in Table 1 that a decrease in *agri* relates to the abandonment of traditional activities in rural areas that increases fuel loads available to burn. Besides, *FAI* was included with time for GLM and had a positive trend in the 2000s for Maxent. These controversial relationships with *agri* and *FAI* could be related to confounding factors such as the increase in fuel loads available to burn, but less use of fire for management due to abandonment and regulations that forbid fire during the high wildfire risk season. Consistent with these results, Rodrigues et al. [67] predicted that *FAI* had a large general contribution for Spain but low for the Madrid region. Regarding *NPA*, GLM did not select it but Maxent considered it relevant in the 2000s with a downward trend, indicating a negative relationship with wildfire probability (Fig 3). It agrees with the premise that protection of natural areas contributes to reduce wildfires in Madrid region and it is not related with the theoretical higher wildfire risk associated with the possible social unrest against the restriction of some activities. This can be due to the fact that only 30% of the *NPA* have a management plan to regulate and control specific activities.

Regarding overall fit GLM model performs steadier than Maxent since it provided an AUC of 0.81 for the 1980s and 0.75 for the 2000s whereas it was 0.74 for Maxent in both periods. This prediction accuracy determines the model ability to estimate the spatial distribution of wildfire occurrence associated to socio-economic drivers. Including a single period of 16 years, Bar Massada et al. [16] obtained better results for Maxent (AUC = 0.72) than for GLM (AUC = 0.66) to predict also wildfire occurrence with a combination of natural and socio-economic drivers. They found differences in the spatial distribution between model outputs, but their Pearson correlation was higher (r = 0.73) than the results of this study for either the 1980s (r = 0.38) or the 2000s (r = 0.4).

Commission error was lower for Maxent than for GLM in all cases. GLM considers presence and absence cells to build the model. Therefore, the model found closer conditions to presence than absence for many true absence cells. Meanwhile, Maxent focuses on presences only and treats absences as background. Consequently, this model did not find as many presences within the true absence cells. One limitation of Maxent could be that the sample might be bias towards some areas in the study site with presences, whereas GLM contains both presence and absence data [59, 68]. Parisien et al. [23] claimed that presence-only models are better where a large portion of the territory does not burn even though is very likely to burn, such as an area with few large wildfires in a short time study window. In relation to the presence-absence models, Bar Massada et al. [16] mentioned that are more justified where only a small portion of the territory is likely to burn, such as an area with parts that never burn for a long wildfire record. This way is more feasible that absences are actually true ones. Furthermore, socio-economic drivers do not follow a known a priori distribution, so non-parametric models like Maxent might perform better than GLM.

To assure the wildfires spatial location, this work combined burnt area from remote sensing and statistical information. Maxent provided a higher sensitivity together with a lower commission error. Instead, the inclusion of the offset term in GLM tends to decrease the predicted probability. The reason for this is that the model was calculated with a zero-sample and then applied to the whole study area using a subset of the absence cells in order to balance the dataset. However, the probability trend depends on the quality of the predictors and not on this offset.

Given that human activities cause ~90% of wildfires in this region, this work focused only on socio-economic drivers and did not consider natural predictors like meteorological data, topographic conditions or fuel models [56, 69]. Previous studies have incorporated these predictor variables. For example, Padilla and Vega-García [66] applied logistic regression models in 2002–2005 to 53 eco-regions in Spain. They included predictors such as distance to towns, *pop* and *roads* to obtain AUC values ranging from 0.74 to 0.95. Besides, Parisien et al. [56] compared models with and without socio-economic drivers to find that the ones that did include them achieved higher AUC. Based on the Western US, their model determined *pop* and *roads* as their key predictors.

## Conclusions

Madrid region represents an example of the socio-economic changes that occurred in relation to wildfire occurrence in the last decades throughout the European Mediterranean basin. By modeling wildfire occurrence in two separated periods, this study explained wildfire occurrence identifying these changes in the socio-economic drivers as part of the exodus from rural to urban areas. Predictors like *pop*, *WUI* and *roads* increased their relevance in the 2000s whereas *FGI* decreased dramatically for both models. Either model adjusted better wildfire occurrence in the 2000s than in the 1980s. Maxent model outperformed GLM in both periods according to indicators like sensitivity or commission error. A steadier result assures the model replicability for other time periods that can help taking preventive measures to manage wildfires in this region. For example, allocate extinction resources in areas with high estimated probabilities, especially those with high ecological value or socio-economic vulnerability.

## Supporting Information

S1 File Socio-economic dataset. S1 File contains data on the independent and dependent variables used for the analysis for the study periods (1980s and 2000s) by 1*1 km grid cell.
(XLS)
